# MutaScope: a high-sensitivity variant caller dedicated to high-throughput PCR amplicons sequencing

**DOI:** 10.1186/1753-6561-6-S6-O22

**Published:** 2012-10-01

**Authors:** Shawn Yost, Hakan Alakus, Hiroko Matsui, Kristen Jepsen, Richard Schwab, Kelly Frazer, Olivier Harismendy

**Affiliations:** 1 Bioinformatics and Systems Biology Graduate program, University of California, San Diego, La Jolla, CA, USA; 2 Division of Genome information Sciences, Department of Pediatrics and Rady Children's Hospital, University of California, San Diego, La Jolla, CA, USA; 3 Moores UCSD Cancer Center, La Jolla, CA, USA; 4 Department of General, Visceral and Cancer Surgery, Center for Integrated Oncology, University of Cologne, Germany; 5 Clinical and Translational Research Institute, University of California San Diego, La Jolla, CA, USA; 6 Institute for Genomic Medicine, University of California San Diego, La Jolla, CA, USA

## 

With the progress of genomics and targeted therapies, an increasing number of cancer somatic mutations are becoming clinically actionable: predictive of drug sensitivity or resistance. However, clinical samples are often suboptimal for their comprehensive detection. Indeed, contamination with normal cells or the presence of diverse subclones affects their detection by high-throughput sequencing. Ultra-deep targeted sequencing (UDT-Seq) is an assay combining microdroplet PCR amplification of exonic sequences followed by a direct, oriented sequencing at high depth of coverage, therefore allowing the detection of low prevalence mutations [1]. Standard sequencing analysis tools that were developed to analyze whole genome and exome shotgun sequencing do not take advantage of UDT-Seq's specific design where each sequencing read originates from a known strand and location. We propose a complete analysis package (MutaScope) dedicated to the analysis of UDT-Seq or similar PCR-based high-throughput sequencing. After alignment to the genome, MutaScope separates the sequencing reads with respect to the amplicons and strand of origin. This allows the experimental measurement of an error rate along the amplicons, which is used to calculate a variant likelihood and rank candidate mutations. Using a set of reference samples, or matched normal DNA, MutaScope then identifies germline and somatic variants and reports them in a unified expanded variant call format. The performance of MutaScope was evaluated on 676 amplicons using a set of calibration samples harboring variants at defined prevalence down to 1%. Overall, MutaScope's sensitivity and positive predictive value (PPV) were >96% and >75%, respectively; which is higher than the standard variant calling strategies (approximately 70% and 70%, respectively). MutaScope detects more than 73% of the variants with an alternate allele frequency ≤5%, while the other methods only detect 30% of the variants. MutaScope offers an analysis strategy specifically dedicated to the identification of low prevalence somatic mutations in high-throughput direct sequencing of PCR amplicons. As a result MutaScope increases the overall technical performance of such approaches that are currently being implemented in clinical diagnostics laboratories.

## References

[B1] Harismendy O Schwab RB Bao L Olson J Rozenzhak S Kotsopoulos SK Pond S Crain B Chee MS Messer K Link DR Frazer KA Detection of low prevalence somatic mutations in solid tumors with ultra-deep targeted sequencing Genome Biol 2011 12 R124 10.1186/gb-2011-12-12-r124 22185227 PMC3334619

